# Community-acquired necrotizing pneumonia due to methicillin-sensitive *Staphylococcus aureus *secreting Panton-Valentine leukocidin: a review of case reports

**DOI:** 10.1186/2110-5820-1-52

**Published:** 2011-12-22

**Authors:** Lukas Kreienbuehl, Emmanuel Charbonney, Philippe Eggimann

**Affiliations:** 1 Department of Anaesthesiology, Hôpitaux Universitaires de Genève (HUG), Geneva, Switzerland; 2 Keenan Research Centre, Li Ka Shing Knowledge Institute St. Michael's Hospital, Toronto, Canada; 3 Department of Intensive Care, Centre Hospitalier Universitaire Vaudois (CHUV), and University of Lausanne, Switzerland

## Abstract

**Background:**

Community-acquired necrotizing pneumonia caused by Panton-Valentine leukocidin (PVL)-secreting *Staphylococcus aureus *is a highly lethal infection that mainly affects healthy children and young adults. Both methicillin-sensitive *S. aureus *(MSSA) and methicillin-resistant *S. aureus *(MRSA) may carry the PVL-phage, but the majority of publications relate to community-associated methicillin-resistant *S. aureus *(CA-MRSA) or mixed patient groups. This study focuses on necrotizing pneumonia due to methicillin-sensitive *S. aureus *strains, with the purpose to determine factors associated with outcome.

**Methods:**

We report a patient with PVL secreting MSSA necrotizing pneumonia and performed a systematic review of similar case in the literature. We analyzed factors associated with outcome.

**Results:**

A total of 32 patient descriptions were retained for analysis. Septic shock (*p *= 0.007), influenza-like prodrome (*p *= 0.02), and the absence of a previous skin and soft-tissue infection (*p *= 0.024) were associated with fatal outcome. In multivariate analysis, influenza-like prodrome (odds ratio (OR), 7.44; 95% confidence interval (CI), 1.24-44.76; *p *= 0.028) and absence of previous skin and soft-tissue infection (OR, 0.09; 95% CI, 0.01-0.86; *p *= 0.036) remained significant predictors of death.

**Conclusions:**

Influenza-like prodrome may be predictive of adverse outcome in PVL-secreting MSSA necrotizing pneumonia. In contrast, previous skin and soft-tissue infection may be associated with improved prognosis.

## Background

*Staphylococcus aureus *is estimated to cause 1-10% of community acquired pneumonias (CAP) and 20-50% of nosocomial pneumonias [1]. It is an important factor of influenza-related morbidity and mortality and approximately half of the patients with *S. aureus *pneumonia have underlying comorbidities and risk factors [2,3]. In 1999, Lina et al. found an association between necrotizing pneumonia and Panton-Valentine leukocidin (PVL)-secreting *S. aureus *[4]. In 2002, Gillet et al. defined the clinical features of PVL-associated necrotizing pneumonia, followed in 2007 by the description of risk factors associated with mortality [5,6]. PVL is thought to be a key factor in the pathogenesis of necrotizing pneumonia. It forms pores in the cell and mitochondrial membrane of neutrophils and macrophages and thus provokes cell lysis and apoptosis with subsequent liberation of inflammatory mediators [4,7]. Some authors contest the pathogenic potential of PVL and suggest the presence of PVL-genes to be a marker of other virulence determinants [8,9].

The global distribution of PVL-carrying *S. aureus *varies geographically. In North America, the most dominant clone is ST8-USA300, which is responsible for the majority of community-associated methicillin-resistant *S. aureus *MRSA (CA-MRSA)-related infections [10,11]. European isolates are more commonly methicillin-sensitive *S. aureus *(MSSA) [4,6]. Overall, the prevalence of PVL-carrying *S. aureus *seems to be increasing. A U.S. wide study examining the proportion of CA-MRSA among *S. aureus *CAP during the 2006-2007 influenza seasons found a prevalence of 79%, in contrast to 12% between 1986 and 2005 [3]. The Health Protection Agency Staphylococcus Reference Unit (HPA-SRU) in England recorded a steady increase of PVL-positive *S. aureus *between 2005 and 2009, with a majority of strains being methicillin-sensitive (61.5% versus 38.5% in 2010) [12]. Molecular profiles of methicillin-sensitive and methicillin-resistant PVL-carrying *S. aureus *reveal close genetic similarity and the former are thought to constitute a reservoir for the latter [13].

Current knowledge about clinical features and mortality of PVL-positive *S. aureus *necrotizing pneumonia is based on series and case reports. The typical clinical picture is a previously healthy child or young adult with an influenza-like prodrome, who rapidly develops septic shock and respiratory failure, in the context of multilobar consolidation, pleural effusion, and airway hemorrhage [5]. Influenza-like prodrome, leuko- and thrombocytopenia, airway hemorrhage, and pleural effusion are considered predictive of fatal outcome [6]. Published mortality rates vary between 40% and 60% [3,6,14,15]. One study compared outcome between MSSA and MRSA strains, without finding a significant difference [14].

We report a patient with PVL-secreting MSSA-necrotizing pneumonia, who had a classical clinical presentation and was successfully treated with antitoxin antibiotics and intravenous immunoglobulin. He was included in a review and analysis of clinical characteristics of reported patients with a PLV-positive methicillin-sensitive *S. aureus *necrotizing pneumonia, with the goal to confirm outcome factors.

## Methods

We searched for case reports and case series about PLV-positive MSSA-community-acquired pneumonias published before April 2010, using PubMed, with the search terms "community-acquired pneumonia," "necrotizing pneumonia," and "Panton-Valentin leukocidin." The reference sections of case reports, case series, and relevant research and review articles were scanned for missed case reports and case series. Case series, which lacked individual clinical patient descriptions, were excluded. Only articles in English, French, and German were analyzed. The patient treated in our own institution was included in the analysis. The extracted clinical, microbiological, and outcome data were converted into variables and analyzed accordingly. For continuous variables, results are summarized as mean ± SD and categorical variables are expressed in proportions. Fisher's exact test and Student's *t *test were used for categorical and continuous variables, respectively. Variables significantly associated with outcome in the univariate analysis were included in a multivariable model. For all tests, a two-tailed *P *value < 0.05 was considered to denote statistical significance. Data analysis was performed with SAS 9.2 (SAS Institute Inc.: Cary, NC, USA).

### Case report

**Figure 1:**
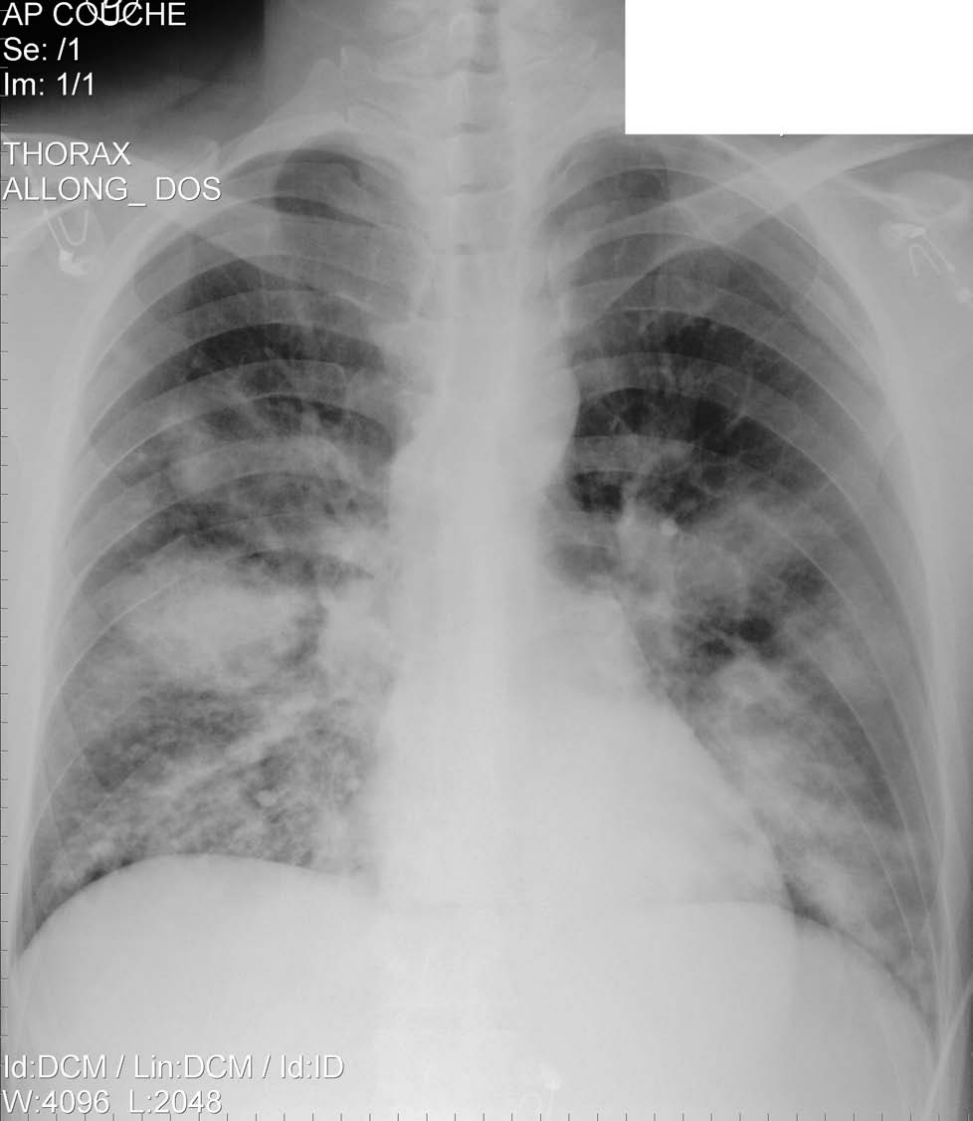
**Chest radiograph on admission showing bilateral dense infiltrates**.

**Figure 2:**
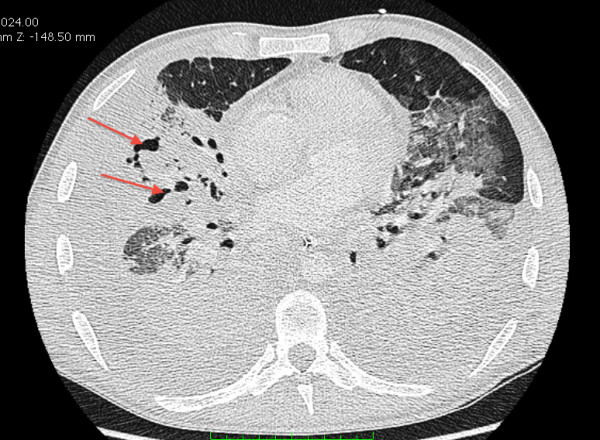
**Lung CT scan on day 12 of hospitalization showing abscess formations in the right middle lobe (arrows)**.

A 32-year-old, previously healthy, Caucasian male presented with severe sepsis and acute respiratory failure. In the previous week, he noted an influenza-like illness. On examination, chest auscultation revealed discrete inspiratory crackles over the lower lung fields. The chest radiograph showed bilateral dense alveolo-interstitial infiltrates predominant in the middle and lower lobes (Figure 1). The leukocyte count was 2.8 G/l, with a left shift of 38%. CRP was 193 mg/l. Other laboratory parameters were in the normal range. Because of a penicillin allergy, the patient was started on levofloxacin. Within the first 24 hours, hypoxemia worsened (PaO_2_/FiO_2 _< 100 mmHg), profound septic shock developed, and the leukocyte count dropped to 0.9 G/l. Sheets of gram-positive cocci on sputum stain prompted the addition of vancomycin. A sputum culture grew MSSA. The HIV test was negative. Polymerase chain reaction (PCR) performed on a throat swab was positive for influenza B (880 cp/ml). The patient remained febrile and a CT scan on the third day revealed extensive infiltrations with cavitations suggestive of multiple abscesses. Suspecting a PVL toxin-secreting strain, antibiotherapy was switched to clindamycin (600 mg qid) and linezolid (600 mg bid) to downregulate the production of the toxin. High-dose intravenous immunoglobulin (2 g/kg) was added for 2 days. Within the following 48 hours, fever decreased with marked improvement of the patient's clinical condition and inflammatory parameters. Further characterization of the *S. aureus *strain confirmed PVL production. Despite rapid initial improvement, the patient required prolonged mechanical ventilation and antibiotherapy because of abscess development and several episodes of acute respiratory distress after proximal bronchi obstruction with plugs of necrotic lung tissue (Figure 2). The total duration of clindamycin and linezolid treatment was 29 and 34 days, respectively. The patient was successfully weaned after 31 days of mechanical ventilation and transferred to the medical ward after 38 days in the intensive care unit. He was discharged from the hospital after 50 days. On follow-up 1 year later, he showed residual dyspnea with heavy exertion but was working again full-time.

## Results

The literature search for MSSA PVL-positive CAP resulted in 31 patient descriptions out of 25 publications [5,6,10,13-40]. Twenty-one publications reported European patients, 14 of which were from France. Six publications originated in the United States, three in Asia, and one in Australia. Cases occurred between 1998 and 2009. Most case reports lack detailed data on history, clinical, and laboratory characteristics. Table 1 lists the variables, which were reported often enough to be included in the analysis. Although 93% of patients (26/28) had multilobar pulmonary involvement and were likely to have ARDS, this diagnosis was not used as a variable because of missing blood gas results and pulmonary wedge pressures.

**Table 1:** Univariate analysis of risk factors associated with mortality in patients with PVL-secreting MSSA-necrotizing CAP

	Died (N = 13)	Survived (N = 19)	Univariate analysis OR (95% CI)	*P *value
**Demographics**				

Age (yr), mean ± SD	25.6 ± 15.5	23.7 ± 17.2		0.752

Male gender	6/13 (46%)	14/19 (74%)	0.31 (0.07-1.36)	0.15

**Clinical characteristics**				

Influenza-like prodrome^a^	9/12 (75%)	4/16 (25%)	9.00 (1.60-50.7)	0.02

Confirmed influenza coinfection	0/3	3/4 (75%)	0.06 (0.002-2.08)	0.143

SSTI on admission	1/13 (8%)	9/19 (47%)	0.09 (0.01-0.86)	0.024

T° < 36° or > 38° on admission	7/11 (63%)	9/11 (82%)	0.39 (0.05-2.77)	0.635

Multilobar involvement	12/12	14/16 (87%)	4.31 (0.19-98.6)	0.492

Lower airway hemorrhage^b^	11/12 (92%)	9/16 (56%)	8.56 (0.88-83.1)	0.088

Septic shock	11/11	7/15 (47%)	26.0 (1.30-522)	0.007

**Laboratory findings**				

Leukocytopenia	9/11 (82%)	8/17 (47%)	5.06 (0.83-30.8)	0.115

Thrombocytopenia	2/8 (25%)	6/6	0.03 (0.001-0.75)	0.01

Coagulopathy	9/9	6/8 (75%)	7.31 (0.30-178.7)	0.206

Positive blood cultures	5/13 (38%)	8/17 (47%)	0.56 (0.13-2.41)	0.484

**Treatment**				

Mechanical ventilation	11/12 (92%)	10/14 (71%)	10.7 (0.52-223)	0.33

First-line antibiotics targeting toxin production^c^	0/12	0/18	--	1

Intravenous IgG	1/13 (8%)	5/19 (26%)	0.26 (0.03-2.51)	0.361

^a^Influenza-like syndrome > 48 h before admission. Symptoms include fever, shivering, chills, malaise, dry cough, loss of appetite, body aches, and nausea.

^b^Hemoptysis and/or macroscopic blood on bronchoscopy/BAL.

^c^Clindamycin, linezolid, or rifampicin,

PVL, Panton-Valentine leukocidin; MSSA, methicillin-sensitive *Staphylococcus aureus*; CAP, community-acquired pneumonia; SSTI, skin and soft-tissue infection.

The average age was 24.5 (interquartile range, 14-38) years, and 13 patients died (41%). With the exception of one patient who died after 20 days, the median time from admission to death was 20 hours. Univariate analysis found that influenza-like prodrome (*p *= 0.02), absence of skin and soft tissue infection (SSTI) on admission (*p *= 0.024), and septic shock (*p *= 0.007) were associated with death (Table 1). The multivariable model confirmed an association with fatal outcome for influenza-like prodrome (OR, 7.44; 95% CI, 1.24-44.76; *p *= 0.028) and absent SSTI (OR, 0.09; 95% CI, 0.01-0.86; *p *= 0.036). Among patients with SSTI, there was a lower rate of preceding influenza-like syndrome (*p *= 0.0008), septic shock (*p *= 0.014), mechanical ventilation (*p *= 0.047), and lower mortality (*p *= 0.024). None of the patients received an initial antibiotherapy targeting the PVL toxin production.

## Discussion

Community-acquired necrotizing pneumonia due to *S. aureus-*secreting PLV toxin is a highly lethal infection, affecting a young and healthy population group [5]. The hallmarks are an influenza-like prodrome, leukopenia, rapid progression to septic shock, and respiratory distress, with multilobar necrosis and haemoptysis [5,6,14].

In this series, the mortality rate was 41%, which is lower than most of previously published rates [3,5,14,15]. On multivariable analysis, influenza-like prodrome predicted fatal outcome. The true proportion of influenza infection is difficult to assess, because influenza testing is not routinely performed and rapid test sensitivity is only 50-70% [41]. Influenza impedes phagocytic killing and damages the trachea-bronchial epithelium with subsequent impairment of secretion clearance and facilitated bacterial adhesion [42-44]. The influenza-like prodrome also may be caused by other respiratory viruses or by *S. aureus *itself.

We found a significant reduction of mortality for patients with skin and soft-tissue infection on admission. This result is new in the context of PVL-associated *S. aureus *necrotizing pneumonia, although one study mentioned a nonsignificant trend toward lower mortality for patients with a history of furuncles [6] and a recent retrospective study found a significant protective effect of a history of PVL-associated infections [45]. Similar results also have been published in studies on *S. aureus *carriers. Approximately 20-30% of healthy persons are persistently colonized with *S. aureus *[44,46]. When hospitalized, these carriers have an increased risk of developing severe *S. aureus *infection caused by their colonizing strain [47], but mortality of *S. aureus *bacteremia is much lower in carriers than in noncarriers [48]. The likely explanation for this protective effect is the stimulation of an immune response, which lowers the intensity of a subsequent invasive infection [49]. A PVL vaccine has already been successfully tested on mice models and may find a human application in the near future [50].

Another issue raised by this study is the high rate of inadequate initial antibiotic treatment regimens. None of the 32 published cases received an antibiotic with an antitoxin effect as part of their first-line treatment, and all but three patients received beta-lactams. The use of beta-lactams as first-line treatment is controversial, because drug levels below the minimum inhibitory concentration may increase toxin release and stimulate toxin production [37,38,51]. The former effect is due to release of intracellular toxin secondary to cell wall lysis. In vivo, low drug levels in target tissues are a consequence of extensive tissue necrosis, leading to poor antibiotic diffusion and a sepsis-related increase of the volume of distribution. However, the stimulatory effect on toxin release is reversed when beta-lactams are given in association with clindamycin, linezolid, or rifampicin [52]. The high rate of inadequate initial antibiotic treatment may be explained by the low prevalence of necrotizing *S. aureus *pneumonia, the low specificity of initial clinical signs and symptoms, and the adherence to treatment guidelines for community-acquired pneumonias. However, even after overt clinical suspicion or microbiological confirmation of PVL-secreting *S. aureus*, only 36% (5/14) of second-line antibiotics were adequate. Not surprisingly, the rate of adequacy was higher among more recent case reports. Since 2007, the Infectious Diseases Society of America (IDSA) recommends adding vancomycin or linezolid in case of severe pneumonia due to CA-MRSA [53]. In the United Kingdom, the Health Protection Agency (HPA) recommends a combination of clindamycin, linezolid, and rifampicin but explicitly dissuades from the use of beta-lactams [54]. Based on the discussed in vitro findings for beta-lactams, a recent recommendation by Gillet et al. suggests a third-generation cephalosporin with vancomycin and clindamycin or linezolid as first-line antibiotherapy. In the case of MSSA, vancomycin can be replaced by oxacillin [55].

Intravenous immunoglobulin may be an important adjunct to antibiotherapy. As illustrated in the above and in other case reports, it has been used successfully on a sporadic basis [30,36,56]. It was studied in vitro and was shown to neutralize PVL-induced pore formation and cytopathic effect [57]. The HPA recommends intravenous immunoglobulin at a dose of 2 g/kg to be repeated after 48 hours if there is persistence of septic shock or failure to respond [54].

The significance of our study results is limited by its reliance on a relatively small number of case reports. Also, many variables, such as thrombocytopenia or kidney function, were reported infrequently and thus could not be included in the statistical analysis. We suspect that only the most severe cases of CAP caused by PVL-secreting MSSA are reported and that many cases are not detected, making it difficult to describe the full spectrum of clinical illness and to form meaningful conclusions based on the case reports. To improve our knowledge of the epidemiology, diagnosis, and treatment, there is a need to establish an international database.

## Conclusions

Necrotizing pneumonia due to PVL-secreting *S. aureus *mandates prompt recognition and specific treatment to prevent premature death in immunocompetent patients. Early suspicion should be triggered by the presence of influenza-like prodrome, leucopenia, rapid progression to septic shock, respiratory distress with multilobar necrosis, and hemoptysis. For PVL-secreting MSSA-necrotizing pneumonia, influenza-like prodrome may be associated with fatal outcome, whereas previous SSTI may reduce mortality. Further studies based on a larger patient number are necessary to confirm this finding.

## Competing interests

The authors declare that they have no competing interests.

## Authors' contributions

LK and PE conceived the study and wrote the manuscript. EC provided data statistics and participated in data interpretation and final writing. All authors read and approved the final manuscript.
